# The effects of evidence-based expert testimony on perceptions of child sexual abuse involving recantation

**DOI:** 10.1371/journal.pone.0254961

**Published:** 2021-08-05

**Authors:** Emily Denne, Stacia N. Stolzenberg, Tess M. S. Neal

**Affiliations:** 1 New College of Interdisciplinary Arts & Sciences, Arizona State University, Glendale, Arizona, United States of America; 2 Criminology and Criminal Justice, Arizona State University, Phoenix, Arizona, United States of America; University of Padova, ITALY

## Abstract

Child sexual abuse (CSA) cases involving recantation invoke concerns about children’s reliability. Expert testimony can help explain the complexities of these cases. Experts have historically relied on Child Sexual Abuse Accommodation Syndrome (CSAAS), yet this is not science-based. In a CSA case involving recantation, how would evidence-based testimony affect perceptions of child credibility when compared to CSAAS? Across 2 studies, we test the effects of expert testimony based on evidence-based science, nonscientific evidence, and experience-based evidence on outcomes in CSA cases involving recantation. Evidence-based testimony led to higher perceptions of credibility and scientific rigor of the evidence when compared to CSAAS testimony. Evidence-based testimony also led to more guilty verdicts when compared to the control. In sum, jurors had some ability to detect evidence strength, such that evidence-based expert testimony was superior to CSAAS testimony in many respects, and consistently superior to experience-based testimony in these cases.

## Introduction

In a 2015 Kentucky court case, a youth minister was charged with first-degree sodomy and first-degree sexual assault of a child. The child did not immediately disclose the assault to his mother, but instead waited 5 days. A detective was brought in as an expert witness and testified regarding Child Sexual Abuse Accommodation Syndrome (CSAAS), arguing that delayed disclosure did not suggest a report is unreliable. The jury convicted the defendant on all charges. The defendant then appealed the use of CSAAS, and upon review the CSAAS expert testimony evidence was not found to meet the rigorous legal criteria of admissible evidence [1, 2]. In a similar case, CSAAS evidence was ruled no longer admissible as evidence in New Jersey courts when used to support denial of abuse or recantation [3].

Research on child sexual abuse (CSA), as well as on questioning CSA victims, has advanced substantially in recent years [4]. Yet, it appears that jurors and experts alike lack a firm understanding of the dynamics of CSA and the process by which children disclose abuse. This seems to be particularly true when a child recants an allegation of CSA, leading to perhaps fewer prosecutions of cases involving recantation than might be warranted [5]. Of course, not all cases involving recantation should be prosecuted, as some may include false reports of abuse; yet, there is research suggesting that at least a proportion of cases where children recant involve true allegations of abuse [e.g., 6]. Failure to prosecute legitimate cases of CSA is a problem, not least because children who recant often lack supportive home environments [7], leaving these children and other children in the home vulnerable to future abuse. In cases where prosecutors choose to proceed, how can they establish child credibility when a child recants CSA allegations? Importantly, how can expert testimony establish whether a child is trustworthy and believable as a witness in CSA cases involving a recantation?.

### Child sexual abuse accommodation syndrome

In 1983, Summit released an influential paper in which he identified Child Sexual Abuse Accommodation Syndrome, describing CSAAS as the process by which children disclose intrafamilial sexual abuse. The CSAAS model described the disclosure process as it unfolds in a particular, predictable way over time. According to Summit’s theory of CSAAS, disclosure involved feelings of helplessness, entrapment, large delays in disclosure, denial of abuse, and recantation of abuse for the vast majority of children [8]. Summit’s paper made bold claims about the supposed mechanisms of abuse disclosure for children who are true victims. Summit’s paper was one of the most influential in the field of CSA disclosure. It was even used by some as a diagnostic tool to detect CSA, despite Summit’s refutation of his paper for that use [9]. Summit’s development of CSAAS was based entirely on a clinical framework. Yet, many of these dynamics of abuse have been supported by researchers since CSAAS was proposed [10–12]. However, there is no one specific disclosure process exhibited by the majority of children, suggesting models like CSAAS may be unreliable [13].

### Recantation in CSA cases

Despite evidence which suggests children can serve as reliable (i.e. consistent) witnesses [14], people tend to believe, accurately, that children are less reliable than adults [15]. Indeed, children are developmentally less mature than adults, making them more susceptible to coaching and the influence of leading questions [16]. Since a victim’s testimony is the main, if not the only source of evidence in many CSA cases, a lot of weight is given to evaluating child credibility (i.e. trustworthiness) [17]. Unfortunately, researchers find that jury eligible participants vary greatly in their understanding of child reliability, credibility, and competence as witnesses [18].

Children’s credibility and reliability refers to their ability to serve as consistent, competent witnesses whose narratives are believable. Children’s narratives are particularly important in CSA cases as there is often a lack of physical evidence to corroborate a child’s claim. Much of a child’s credibility is then dependent on both their memories of events and their ability to communicate those memories [19]. The ways in which we question children about memories of abuse are strong predictors of their ability to communicate those memoires. For example, researchers find that open-invitations yield the most productive reports of abuse from children [20]. While children’s narratives form the foundation of their credibility and reliability, additional evidence in the case, such as confirmatory or disconformity evidence, can influence assessments. As such, some cases contain strong confirmatory evidence such as a perpetrator confession, and other contain evidence that jurors may question, such as recantation [21, 22]. Jurors and experts alike are skeptical about the credibility of children in abuse allegation cases [23], especially when children recant abuse allegations [see e.g., 24]. Recantation rates can be difficult to interpret as they point to one of two explanations: either the child was correcting an untrue statement or making a false denial [25]. The literature on recantation is limited and is the source of an important debate within the field [cf. 7, 25]; however, studies suggest that recantation does not necessarily indicate that the child made a false allegation [see e.g. 26, 27].

If recantation signals a false abuse allegation, then rates of recantation should presumably be higher in these cases as compared to abuse allegation cases with corroborative evidence (e.g., medical evidence, perpetrator admission, and multiple victims). However, high rates of recantation are seen both in cases with and without corroborative evidence, with rates ranging from 23% to 50% [7, 28]. Specifically, Gordan and Jaudes (1996) conducted a case review of 14 children who had corroborative evidence of CSA (a sexually transmitted disease). Of those children in the study who disclosed CSA, 50% later recanted despite very strong evidence suggesting they were CSA victims. Malloy, Lyon, and Quas (2007) found that “high-risk” children, or those who are young, have low maternal support, and stay in contact with their alleged abuser are more likely to recant.

Of course, some recantations may be reflections of false allegations. Take for example, findings from Gonzalez and colleagues (1993), who noted recantation rates of 27% in their sample of 63 children who reported satanic and ritual abuse in a preschool. These cases notably involved suggestive questioning resulting in false allegations. While a large body of research suggests that recantations are not necessarily indicative of false accusations, children can and do make false accusations and may then set the record straight by taking back their original allegation [29]. Simply put, based on the sole evidence that a child recants, we do not conclusively know which allegations are true or false and whether the recantation is being used to correct an untrue allegation or mask a true one. Importantly, since we do not know, recantations should be given careful evaluation and should not be used to immediately discredit a victim. Still these cases are particularly complicated to prosecute.

While the literature is limited, and sometimes the samples quite small, the evidence thus far supports the notion that CSA recantation may be part of the process of true disclosure for *some* children. It is these cases where prosecutors may wish to proceed, but may be concerned about how to address children’s credibility, and whether children will be believed.

### The need for expert testimony in CSA cases

How calibrated are jurors to the strength and validity of the evidence being presented to them? Some researchers suggest that jurors tend to be poor at distinguishing between weak and strong scientific evidence [30, 31]. Alternatively, some researchers suggest that jurors have some ability to distinguish between weak and strong evidence [32, 33]. For example, Smith and colleagues (2011) explored the effects of strong and weak evidence with and without case context to determine how jurors are calibrated to evidence strength. Jurors were able to distinguish weak evidence from strong evidence when presented alone. However, participants were less able to distinguish strong and weak evidence when presented in the context of a case.

Researchers have consistently supported the need for expert testimony to elucidate the dynamics of CSA and its positive effects on jurors’ comprehension of evidence [34, 35]. Yet, Stolzenberg and Lyon (2014) found that a child abuse expert testified in only 9% of CSA cases, suggesting the prosecution may be infrequently educating jurors on the inconsistent behaviors in child reporting [36]. How best to do this remains unclear. Indeed, jurors’ understanding of CSA is limited and varies widely. For example, jurors tend to be ill-informed about the typical perpetrators of abuse, the extent of physical harm to the child, the frequency of CSA, the most common forms of CSA, the bounds of children’s memory, and the range of children‘s reactions to abuse [37]. While jurors’ understanding of CSA is limited, jurors may have some limited intuitive knowledge of CSA. For example, Quas and colleagues (2005) found that 84% of people believed a child would delay disclosure of CSA [18].

Jurors’ conceptualization of CSA and their endorsement of CSA misconceptions influence their perceptions of the case in important ways. The more CSA misconceptions a juror held, the less they viewed a child victim as a credible witness [34]. Jurors’ conceptions of CSA and children as witness in general tends to be influenced by several juror characteristics including gender. Indeed, women tend to convict the defendant more often in CSA cases than men [38], men tend to view child victims as less credible than women [39], and men view CSA as less severe than women [40]. Expert testimony evidence is thus a critical avenue for attorneys to correct jurors’ misconceptions about CSA.

### The use of expert testimony in CSA cases

Despite the recent rulings which prohibited CSAAS as admissible evidence in New Jersey and Kentucky courts [2, 3], there seems to be a lack of consistency across states regarding the admissibility of such testimony. In a 2014 Arizona case, Martin Salazar-Mercado was charged with child molestation and sexual conduct with a minor under age 15. CSAAS was admitted as evidence in this case; a ruling which held even upon appeal by the defendant [41]. Indeed, this form of syndromal evidence is only ever admissible as a tool to describe children’s behavior and should not be indicative of guilt [1]–but can jurors correctly interpret the evidence presented to them?.

As previous research has demonstrated, jurors have varied greatly in their understanding of child credibility, making the use of expert testimony particularly influential [18]. Yet, CSAAS lacks sufficient facts or data and is not the product of reliable principles and methods [33]. Others have commented on the problem of CSAAS as admissible evidence in court, yet no experimental research has explored how else experts can educate jurors about the dynamics and mechanics of child sexual abuse in these cases [1]. If CSAAS testimony is considered weak evidence, and inadmissible evidence in some states, how else can experts effectively educate jurors about the nonspecific diagnosticity of recantation? This recent inconsistency in the admissibility of CSAAS testimony makes this area of research both timely and important.

Some research has explored the impacts of evidence type in CSA cases. For example, Kovera and colleagues (1994; [42]) conducted a study exploring three types of evidence (syndromal [CSAAS], credibility based, or anatomical doll) in CSA cases to explore how jurors were influenced by such evidence. Kovera found that jurors who were exposed to expert testimony were more likely to convict the defendant than were those not exposed to expert testimony. In addition, jurors were less biased by syndromal evidence (CSAAS evidence) than by credibility or anatomical doll data. Participants who were exposed to syndromal evidence rated the evidence as less important and less helpful than case history data. However, expert testimony-type (syndromal vs. case history data) and perceptions of the scientific basis of the expert’s testimony did not influence participants case verdicts [40]. While this study set the foundation for the present line of research, it left several questions unanswered. Specifically, how does this line of work translate to a recantation case? How does CSAAS compare to other forms of stronger, scientifically-supported evidence? We were interested in adding to the literature about when and how expert testimony might be useful for aiding the jury in understanding evidence about the role of recantation in abuse allegation cases.

## Study 1

Based on recent practices, laws, and the body of research on recantation, we became interested in the effects of expert testimony in cases of child sexual abuse case where children recant. It is clear that children’s credibility is of particular concern in these cases, establishing the central role of expert testimony to encourage judges and jurors to critically evaluate the child’s reliability and credibility rather than automatically dismissing the child’s allegations. Importantly, it appears that a popular form of testimony, CSAAS, lacks scientific rigor and an evidence-based empirical foundation despite modern research which supports many of these original claims. What can experts do to explain the dynamics of CSA and influence perceptions of child credibility in these cases? Does the strength of the evidence presented in expert testimony affect jurors’ perceptions of child credibility and case outcomes when a child recants an allegation of CSA? How does gender influence perception of these cases? The purpose of this study was to establish how calibrated jurors were to evidence strength and how different forms of evidence influenced their perceptions of cases involving recantation of CSA. Specifically, mock jurors reviewed three cases involving recantation and three forms of evidence before providing their perception of the cases and case verdict. This line of work yields concrete and practical recommendations for informing expert testimony and informing law and policy in these sensitive cases. All case materials and hypothesis were preregistered and time-stamped on the Open Science Framework (DOI 10.17605/OSF.IO/F87XT).

### Hypotheses

#### Verdict and confidence in verdict

Based on previous research suggesting perceived evidence strength may influence jurors’ verdicts [30], we expected participants would render a guilty verdict more often and have more confidence in their verdict when presented with evidence-based expert testimony than when presented with CSAAS or the control expert testimony. In addition, participants would render a guilty verdict more often and have more confidence in their verdict in the CSAAS expert testimony condition than in the control expert testimony condition.

#### Defendant blame

We expected that expert testimony would influence perceptions of the defendant such that participants would attribute more blame to the defendant when presented with evidence-based testimony when compared to CSAAS testimony and the control testimony. They would further attribute more blame when presented with CSAAS testimony than when presented with the control testimony.

#### Believability and reliability of the child

We expected expert testimony to influence participants’ perceptions of child believability and reliability such that participants would find the child more believable and reliable when presented with evidence-based expert testimony than in either CSAAS expert testimony or the control testimony. Furthermore, participants would find the child more believable and reliable when presented with CSAAS testimony than when presented with the control expert testimony.

#### Evidence strength and scientific value

In line with research which supports jurors’ abilities to discern weak and evidence-based evidence [33], participants would accurately rate the CSAAS evidence as weaker and less scientific than the evidence-based evidence and would rate the control evidence as weaker than both other forms of evidence.

### Method

#### Participants

We conducted power analyses estimating a small effect size of ꞃ_p_^2^ .01, with an *α* of .05, and 95% power for Likert-scale items yielding a necessary sample size of 259 participants. Our final sample consisted of 270 jury eligible citizens (over the age of 18 who have not committed a felony) recruited online via Amazon’s Mechanical Turk. Ten participants failed all three manipulation checks assessing jurors’ comprehension of expert testimony, or indicated that they failed to understand jury instructions, and were excluded from analyses. Manipulation checks involved a comprehension question given after each of three cases asking jurors about the type of evidence that had been presented to ensure they had read the testimony carefully. Participants ranged in age from 20 to 71 (*M*_age_ = 34.06, *SD* = 9.77); and were predominantly male (62.0%) and White (68.7%; 8.1% Asian; 1.8% Hispanic/Latino, 6.4% African American; 1.3% Native American; 1.7% other).

#### Design

**Fig 1:**
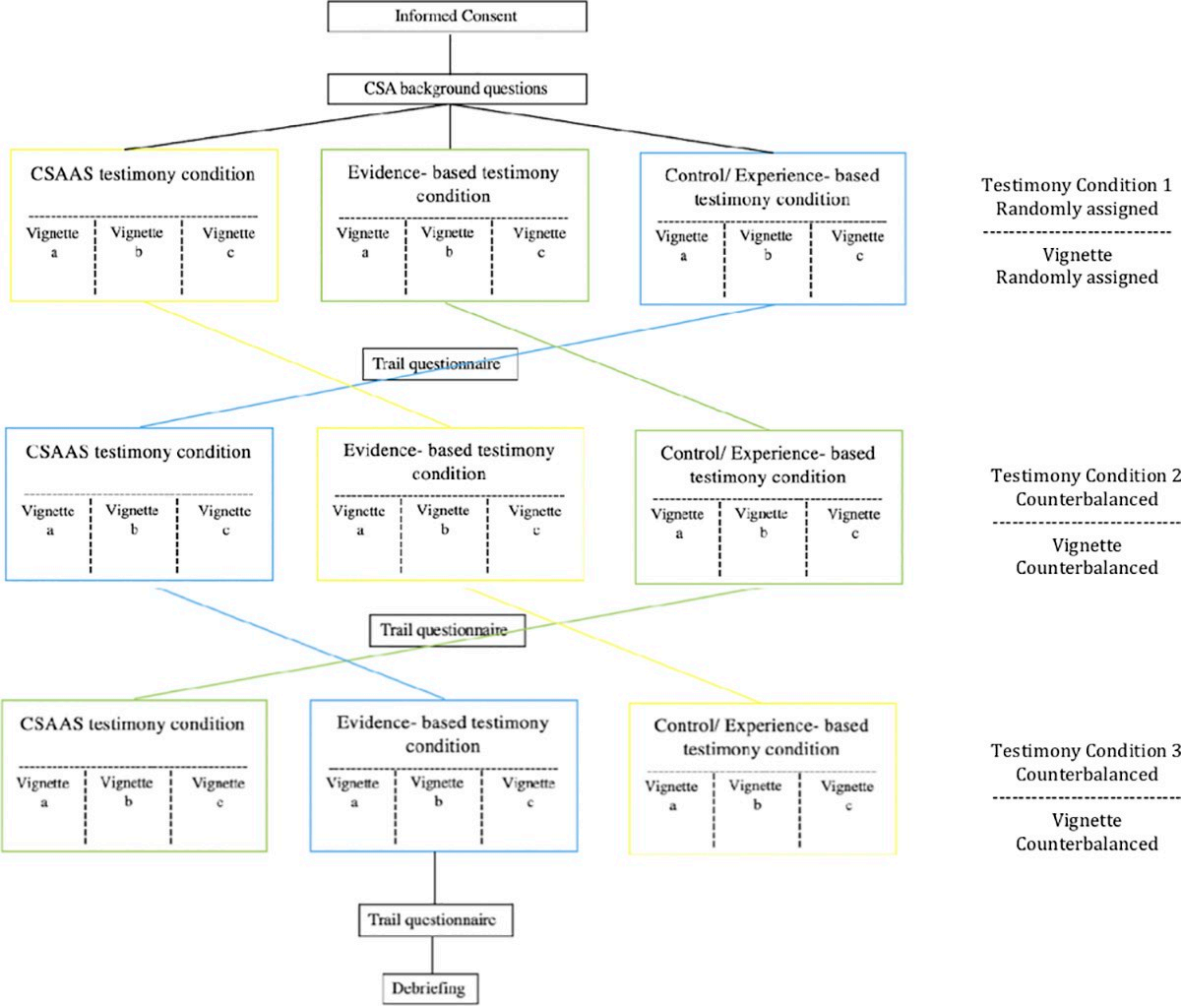
Flow chart for study 1 demonstrating counterbalancing and randomization. The three testimony conditions were counterbalanced, shown in 3 different colors. The three case vignettes (a = fondling, b = exhibitionism, c = pornography) were counterbalanced. The vignettes also included a unique combination of randomly piped in surface-level-details at each instance (i.e. defendant and victim first and last names, age of the victim, race of the victim, etc.).

All materials were approved by Arizona State University’s Institutional Review Board. This study utilized a three-condition within-subjects design to explore the effects of expert testimony on perceptions of the child victim and guilt of the defendant (see Fig 1). While a between-subjects design is the norm in social psychological research, there is convincing rationale in favor of using a within-subjects design. A within-subjects design guards against the problems associated with pre-existing group differences and randomization failure. In addition, it allows for high control and increased statistical power. The error in within-subject’s design is typically smaller when compared to a between-subjects design allowing for a cleaner test of our hypotheses. While carryover effects serve as a natural confound in within-subjects research, we used counterbalancing to reduce these carryover effects [43]. We further randomized piped surface-level details about which we did not care for each case to reduce confounds.

The three counterbalanced conditions included CSA with recantation and a) evidence-based expert testimony, b) CSAAS expert testimony, or c) control (experience-based) testimony. These conditions were carefully developed and reviewed by a clinical psychologist for consistency of information across the sources–we varied only the source of the information but kept the content comparable across the three testimony-types. The content of information for each condition was consistent with the source of the information. For example, across all three conditions the expert addresses disclosure behavior, recantation, and child reactions to CSA. These conditions were developed and amended based on the typical testimony used in recent expert testimony given on CSA in Maricopa County, Arizona.

Evidence-based Expert Testimony. We developed an evidence-based expert testimony condition depicting modern empirical evidence presenting research on recantation. Testimony included statements such as, “There is no definitive way to tell if a child is telling the truth. However, a lot of research has been conducted on just this question. There was a study published by Dr. Lindsay Malloy in 2007 that looked at children who changed their statements in a child sexual abuse case. Basically, she found that about 20% of kids took back an allegation of abuse. Dr. Malloy analyzed that data looking for instances where false allegations are high.” The testimony details why a child might disclose and recant, specific data from Dr. Malloy, empirically supported estimates of recantation rates, as well as how children react to CSA.

Nonscientific Expert Testimony. Our nonscientific expert testimony condition depicted CSAAS testimony describing syndromal evidence. For example: “Yes, so CSAAS is a syndrome proposed by Roland C. Summit in the mid-to-late 1970s. He uses CSAAS to describe how sexually abused children react to abuse. Summit suggests there are 5 stages which children go through in disclosing sexual abuse which I discussed earlier.” The expert describes children’s disclosure behaviors, recantation, and reactions to abuse all within the framework of CSAAS.

Control: Experience-Based Clinical Opinion Expert Testimony. Our control condition depicted clinical experience-based testimony. Our transcript included statements such as: “I make my own mental list of possible indicators of CSA that I look for to help me decide if a child has been abused. I look at their family environment, their age, all of the child’s claims, and the plausibility of their claims. Then based on that I go with my intuition.” Again, this testimony describes children’s disclosure, recantation, and children’s reaction to CSA based entirely on the expert’s clinical experience with no reference to empirical evidence.

#### Procedure and materials

After providing written informed consent, participants answered some general frequency questions about child sexual abuse. We were interested in participants generally understanding about CSA reporting dynamics as this could provide important rational for the use of expert testimony. Participants estimated the frequency of CSA, frequency of victim recantation, and frequency of CSA false allegations ranging from 0% of the time (*never*) to 100% of the time (*always*).

Participants then read one of three summaries of a case of child sexual abuse followed by one of three expert-testimony transcripts. For each condition, participants read a brief summary of a case of child sexual abuse (3 total), each that introduced surface-level case differences to reduce carryover effects, and each containing one of the following: exhibitionism, exposure to pornography, and touching over clothes (see OSF site for materials).

The case descriptions involved a long paragraph describing the child, their allegations against the perpetrator, and disclosure process. It was during the course of the trial, interview, or report that the child recanted all allegations of abuse and claimed none of the events truly happened. The order of case description was randomized and included a randomly piped-in name for the defendant and victim from a list of the seven most popular male names, female names, and last names in the United States. The race of the child (White or Black) was randomly piped-in using a random pipe function in Qualtrics. The perpetrator was always male and the victim always female. Finally, the age of the child was randomly piped-in ranging from 6–14 to vary the case descriptions and control for confounding effects of names, race, or age. We chose these ages as they represent a range of younger and older children. Recantation in all case scenarios was conceptualized as a full retraction of abuse allegations.

Following each case description, participants read one of three abridged trials in which we embedded our manipulated variables of interest. The transcripts involved a case of CSA with recantation and either evidence-based expert testimony, CSAAS expert testimony, or our control condition. In line with legal standards, each teaching expert only addressed the general characteristics of a CSA victim without providing any insight into the specific behavior of the alleged victim.

The expert testimony included 10 question-answer pairs establishing the expert’s credentials, evidence to support the child as potentially credible despite recantation, as well as clear details describing the source of the expert’s claims. This was held constant across conditions. Before asking testimony-specific questions, the expert was briefly asked about their qualifications. To ensure that the expert’s credentials did not influence the impact of their statements, the expert’s occupation was randomly piped in using one of seven occupations related to CSA (e.g., director at West Children’s Hospital, employed by the Arizona Department of Child Safety) as well as years of experience ranging from 12-to-15 to vary the conditions while controlling for confounds related to expertise. We kept this range narrow since experience may be a valuable indicator of expertise.

Finally, participants received a brief summary of the cross-examination of the expert (one paragraph) attacking the expert’s credibility. The summary involved a short paragraph depicting typical arguments given in CSA cases attacking the source of the expert’s evidence. In total, the case description, transcript, and cross examination were two and a half pages long. Participants received all three conditions, which were counterbalanced to control for order effects. In addition, the testimony-type with case description was counterbalanced. Since some CSA cases do not have corroborative evidence [44, 45], we did not provide any additional evidence in the case.

For each case description (3 total), participants used a combination of Likert scales, dichotomous measures, and percentage ranges to complete an evaluation of the guilt of the defendant (*guilty* vs. *not guilty*), confidence in their verdict (*0% - 100%*), blame attributed to the defendant (*1*, *not at all blameworthy to 7*, *completely blameworthy*), believability of the child (*1*, *extremely unbelievable to 7*, *extremely believable*), reliability of the child (*1*, *extremely unreliable to 7*, *extremely reliable*), evidence strength (*1*, *extremely weak to 7*, *extremely evidence-based*), and judgments about the scientific credibility of evidence (*1*, *not at all credible to 7*, *completely credible*). After completion, participants were compensated with $3.50 and read a debriefing statement. Participants took an average of 19 minutes to complete the survey.

### Results

Prior to testing our hypotheses, we began by exploring participants’ understanding of CSA. Across each question we conducted a one-way ANOVA in SPSS 25 to test for gender differences, as gender differences frequently emerge when exploring perceptions of CSA [45]. When asked about the percentage of children who are sexually abused, the mean reported frequency was 22.74%, *SD* = 17.88. No gender differences emerged. When asked about the percentage of time a child is lying when reporting CSA, mean estimates fell at 18.04%, *SD* = 18.58. male jurors thought children lie significantly more often (*M* = 20.16, *SD* = 18.39) than female jurors (*M* = 13.49, *SD* = 18.26), *F*(1, 269) = 7.75, *p* = .006, ꞃ_p_^2^ = .060. Jurors’ estimates of recantation in CSA cases fell at a mean of 30.00%, *SD* = 20.96 with no gender differences.

For all subsequent analyses (verdict, verdict confidence, defendant blame, child believability and reliability, evidence strength, and credibility), Table 1 presents all findings descriptively.

**Table 1:** Descriptive statistics detailing case perceptions and outcomes by condition in Study 1 and Study 2.

	Evidence-based testimony				CSAAS testimony				Control testimony			
	*Study 1*		*Study 2*		*Study 1*		*Study 2*		*Study 1*		*Study 2*	
	*M*	*SD*	*M*	*SD*	*M*	*SD*	*M*	*SD*	*M*	*SD*	*M*	*SD*
Confidence in Verdict	7.35	2.47	7.17	2.30	7.15	2.57	7.54	2.22	6.94	2.63	7.29	2.40
Blame	5.21	1.67	4.82	1.86	4.94	1.76	4.82	1.81	4.81	1.71	4.67	1.71
Child Believable	5.41	1.32	5.54	1.26	5.31	1.36	5.45	1.31	5.16	1.36	5.34	1.35
Child Reliable	4.83	1.53	4.99	1.52	4.75	1.60	4.91	1.48	4.53	1.62	4.74	1.60
Evidence Strength	4.80	1.42	4.99	1.30	4.08	1.63	4.15	1.63	3.01	1.86	3.59	1.72
Evidence Credibility	5.16	1.30	5.36	1.21	4.28	1.56	4.22	1.63	3.27	1.86	3.79	1.68

Note: Confidence in Verdict (*0%-100%*), Blame (*1*, *not at all blameworthy– 7*, *completely blameworthy*), Child Believability (*1*, *extremely unbelievable– 7*, *extremely believable*), Child Reliability (*1*, *extremely unreliable -7*, *extremely reliable*), Evidence Strength (*1*, *extremely weak– 7*, *extremely strong*), Evidence Credibility (*1*, *not at all credible– 7*, *completely credible*).

#### Verdicts and verdict confidence

To assess whether stronger evidence led to more guilty verdicts, we conducted a Generalized Estimates Equations analysis, comparing participant’s verdicts across the 3 conditions. Guilty verdicts were coded as 1 and not guilty verdicts were coded as 0. We uncovered a significant effect of gender *b* = -.70, *SE* = .22, *p* = .005, *95% CI* (-1.13, -0.26) such that women (74%) were significantly more likely to assign a guilty verdict than men (59%). We further uncovered a significant effect of testimony on verdict such that participants were significantly more likely to assign a guilty verdict in the evidence-based condition (69.5%) when compared to the control testimony (55.8%), condition *b* = -.58, *SE* = .13, *p* < .001, *95% CI* (-.828, -.325) as well as in the CSAAS condition (64.9%) when compared to the control testimony condition *b* = -.36, *SE* = .11, *p* = .002, *95% CI* (-.577, -.135). There were no differences between the CSAAS and the evidence-based expert testimony condition.

To explore differences in confidence in these verdicts, we conducted a repeated-measures ANOVA (repeated measure: evidence-based testimony, CSAAS testimony, the control testimony) by gender with a Greenhouse-Gessier correction to correct for violations of sphericity. There was no significant main effect of gender. There was a significant main effect of testimony type on verdict confidence such that the Evidence-based testimony *p =* .*006*, *95% CI*(.10, .8) led to more confidence than the Control testimony. There was no significant interaction.

#### Defendant blame

There was a significant main effect of testimony type on defendant blame such that the Evidence-based testimony *p* < .001, *95% CI* (.16, .71) led to higher attributions of blame than the Control testimony (*M* = 4.81, *SD* = 1.71). Evidence-based testimony also led to higher attributions of blame than CSAAS testimony *p* = .003, *95% CI*(.09, .57). There were no significant differences between CSAAS testimony and the control. We uncovered a main effect of gender on perceptions of perpetrator blame, such that women (*M* = 5.44, *SD* = 1.65) viewed the perpetrator as more blameworthy than men (*M* = 4.78, *SD* = 1.70). There was no statistically significant interaction.

#### Child believability and reliability

We uncovered a significant main effect of gender, such that women (*M* = 5.66, *SD* = 1.23) viewed the child as more believable than men (*M* = 5.13, *SD* = 1.36). Furthermore, we uncovered a significant main effect of testimony-type on perceptions of believability ranging from (1) *extremely unbelievable* to (7) *extremely believable*. Evidence-based expert testimony led to higher ratings of child believability than the control expert testimony *p* = .001, *95% CI (*.12, .55). There were no significant differences between CSAAS and evidence-based expert testimony or CSAAS and the control testimony. These main effects were subsumed by an interaction between gender and testimony-type. To explore this interaction, we split the file by gender and conducted post-hoc tests. There were no significant differences in perceptions of child believability by testimony-type for men, but there were for women. Women viewed both CSAAS, *p* = .005, *95% CI* (-.71, -.10) and evidence-based expert testimony, *p* < .001, *95% CI* (-.94, -.25) as leading to higher ratings of believability than the control expert testimony.

We then explored whether testimony strength would positively correspond with child reliability (measured on a 7-point Likert scale ranging from 1, *completely unreliable* to 7, *completely reliable*). We found a main effect of gender, such that women (*M* = 5.04, *SD* = 1.47) viewed the child as more reliable than men (*M* = 4.54, *SD* = 1.60). We further found a significant main effect of testimony-type on perceptions of child reliability. Evidence-based expert testimony resulted in higher ratings of child reliability than the control expert testimony, *p =* .006, *95% CI (*.07, .54). CSAAS expert testimony also led to higher ratings of child reliability than the control, *p* = .016, *95% CI* (.36, .47). There were no significant differences between CSAAS and evidence-based expert testimony. There were no interactions.

#### Evidence strength and credibility

To gain a better understanding of how jurors are evaluating the evidence and in turn how it may influence their verdict, we assessed whether mock jurors were calibrated to the variations in strength and credibility of expert testimony. There was no significant main effect of gender. We uncovered a significant main effect of testimony-type on perceptions of strength of the evidence. Evidence-based expert testimony was viewed as stronger than CSAAS, *p* < .001, *95% CI* (.44, .99), and the control expert testimony, *p* < .001, *95% CI* (1.58, 2.20). CSAAS expert testimony was viewed as stronger than the control, *p* < .001, 95% *CI* (.89, 1.46). The main effect of gender was subsumed by a two-way interaction of evidence strength and gender. However, post-hoc tests revealed no significant differences between genders. Results trended such that females viewed the CSAAS evidence as stronger than males did, but viewed the control as weaker.

We then assess mock jurors’ perceptions of evidence credibility. There was no significant main effect of gender. We uncovered a significant main effect of testimony-type on perceptions of credibility of the evidence. Evidence-based expert testimony was perceived as more credible than CSAAS, *p* < .001, *95% CI* (.60, 1.14) and the control expert testimony, *p* < .001, *95% CI* (1.67, 2.33). CSAAS expert testimony was seen as more credible than the control expert testimony, *p* < .001, *95% CI* (.86, 1.41). There was a significant gender by testimony-type interaction. However, post-hoc tests revealed no significant differences between genders.

### Discussion

Our findings support those of previous research, such that women were more likely to render a guilty verdict, attribute more blame to the defendant, and view the victim as more believable and reliable [38, 39]. Generally, there were no statistically significant differences in men and women influenced by the form of expert testimony. Gender differences did not emerge consistently in perceptions and application of testimony-type to each case. Overall, jury-eligible participants were able to distinguish between weak and strong expert testimony, rating evidence-based testimony as stronger and more credible than CSAAS. Participants also rated CSAAS as stronger and more credible than the control testimony, yet both CSAAS and our control testimony were based on clinical intuition. In partial support of Smith and colleagues’ findings (2011), it seems that participants have some ability to discern weak and strong evidence.

While jury-eligible participants could distinguish between the three strengths of evidence, these distinctions did not consistently translate into proportionate differences in verdicts, perceptions of the child, or perceptions of the defendant. Strong evidence and CSAAS were more effective than the control testimony in leading to more guilty verdicts, increased child believability, and increased child reliability. However, jury-eligible participants often struggled to translate their perceptions of difference in the strength of the evidence between evidence-based and CSAAS expert testimony, to case level assessments. Mock jurors did not apply CSAAS evidence and evidence-based expert testimony in different ways despite being able to identify that they differ in scientific rigor. This again suggests that mock jurors’ verdicts and perceptions are still influenced by weak research, even when they can identify that the research is indeed weak.

Empirically-supported evidence was the only form of evidence that was consistently and accurately distinguished from experience-based testimony leading to more positive perceptions of the victim and poorer perceptions of the defendant. However, CSAAS still exerted a strong influence on mock jurors’ perceptions of the case. Our research supports that evidence-based testimony emerged as stronger than the control testimony, but not stronger than CSAAS. This finding, suggests that mock jurors appear only somewhat sensitive to differences in its scientific rigor.

Despite evidence from Study 1 that testimony strength had some effect on participants’ application and understanding of evidence in the case, we had several methodological concerns resulting from the within-subjects nature of our design. It was unclear whether participants made case specific judgments based only on the testimony and information available for each case, or if they applied their cumulative knowledge from each subsequent expert testimony to the cases. Furthermore, in order to blind participants to our hypotheses, our vignettes were fairly different from one another. For example, the timing and recipient of the child’s first disclosure differed between vignettes. To eliminate concerns about carryover effects and isolate our manipulation, we replicated our findings using a high-powered, between-subjects design.

## Study 2

Our hypotheses for Study 2 remained the same as those of Study 1, where we sought to replicate the findings in a high-powered between-subjects design. In addition, to better understand the relationship between perceptions of the child and evidence, we conducted an exploratory serial mediation analysis. Based on our Study 1 results, it was highly likely and theoretically logical that perceptions of the evidence were influencing perceptions of the child and subsequent case outcomes. Currently, it is not possible to conduct mediation analyses on a within-subjects design with more than 2 levels of an independent variable. For this reason, we were only able to conduct exploratory mediation for Study 2, inspired by the results of Study 1. Indeed, this serial mediation is useful for better understanding the process and paths by which jurors are applying testimony to their case verdicts.

### Method

#### Participants

Based on power analyses with an estimated small effect size of ꞃ_p_^2^ .01, a Likert DV, with an alpha of .05, and 95% power we needed a sample of 957 participants. Our final sample consisted of 966 jury eligible citizens (over the age of 18 who have not committed a felony) recruited online via Amazon’s Mechanical Turk. Two-hundred and thirty-seven participants of the original sample failed a manipulation checks assessing mock jurors’ comprehension of expert testimony, indicated that they failed to understand the jury instructions, or indicated that they had participated in a similar study. These participants were excluded from analyses. Participants ranged in age from 18 to 77 (*M*_age_ = 38.74, *SD* = 12.52); and were predominantly female (53.9%) and White (73.0% White, 8.2% African American, 6.2% Hispanic/Latino, 6.9% Asian; 1.4% Native American; 1.8% other).

#### Design, materials, and procedure

Our materials and procedures were highly similar to those in Study 1. Participants were randomly assigned to a vignette condition (fondling over clothes, exhibitionism, or exposure to pornography), randomly assigned to a child race (White or Black), randomly assigned to a child age (6, 10, or 14), and randomly assigned to a testimony condition (evidence-based expert testimony, CSAAS testimony, or our control, experience-based testimony). For the purpose of this paper we were only interested in the effect of expert testimony condition on case related decisions. For this reason, we collapsed across child age, race, and vignette condition. Finally, we modified each vignette slightly to standardize the time of recantation, time of disclosure, and disclosure recipient across vignettes.

Participants read the case, a transcript of 20 question-answer pairs detailing the expert’s testimony, a summary of the cross examination of the expert, and then answered a series of questions about the case. Much as in Study 1, participants evaluated the guilt of the defendant, confidence in their verdict, blame attributed to the defendant, believability of the child, reliability of the child, evidence strength, and judgements about the scientific credibility of the evidence. It was unclear whether participants in Study 1 were differentiating between the words “credibility” and “reliability” in a meaningful way. In Study 2, we provided participants with definitions of each of these. Finally, in Study 1, participants were asked about their general perceptions of CSA before answering case specific questions. To eliminate the effects of order on participant’s responses, half of our participants answered these questions before reading about the case and half answered these questions after reading about the case. After completing the study, participants were financially compensated with $0.75 and read a debriefing statement. Participants took an average of 10 minutes to complete the survey.

### Results

Prior to testing our hypotheses, we conducted a series of one-way ANOVAs to explore gender differences in participants’ perceptions of CSA. When asked about the percentage of children who are sexually abused, the mean reported frequency was 26.89%, *SD* = 17.09. Women (*M* = 29.76, *SD* = 17.76) believed children are abused more often than men (*M* = 22.99, *SD* = 15.31), *F*(1, 958) = 38.31, *p* < .001, ꞃ_p_^2^ = .038. When asked about the percentage of time a child is lying when reporting CSA, mean estimates fell at 16.04%, *SD* = 15.49. Male mock jurors thought children lie significantly more (*M* = 18.68, *SD* = 16.49) than female mock jurors (*M* = 14.09, *SD* = 14.41), *F*(1, 958) = 20.97, *p* < .001, ꞃ_p_^2^ = .021. Mock jurors’ estimates of recantation in CSA cases fell at a mean of 34.14%, *SD* = 22.53. Female mock jurors (*M* = 35.82, *SD* = 23.52) thought children recant more than males (*M* = 31.86, *SD* = 20.94), *F*(1, 958) = 7.27, *p* = .001, ꞃ_p_^2^ = .008.

#### Verdict and verdict confidence

**Fig 2:**
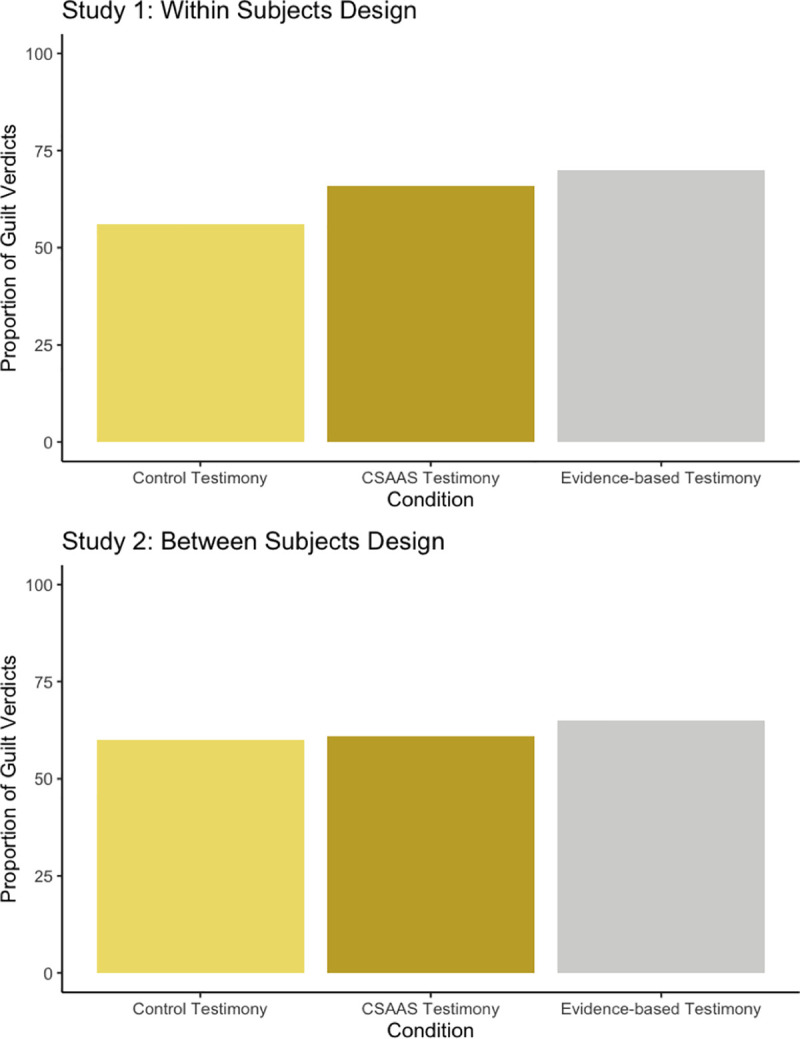
Study 1 and Study 2 proportion of guilt verdicts by condition.

For all subsequent analyses Tables 1 and 2 present all descriptive and inferential findings. We performed a logistic regression to assess the effects of evidence type and gender on likelihood that a participant would offer a guilty verdict (see Fig 2). The model was significant, *X*^2^(1) = 50.40, *p* < .001 and explained 5.3% (Nagelkerke R^2^) of the variance in verdicts, correctly classifying 62.4% of cases. Females (69%) were significantly more like to assign a guilty verdict than males (51%; *B* = -.78, *p* < .001) and evidence-based testimony (65%) led to more guilty verdicts than the control testimony (57%; *B* = -.36, *p* = .038), but CSAAS testimony did not (63%; *B* = .26, *p* = .09). Evidence-based testimony did not lead to more guilty verdicts compared to CSAAS (*B* = -.09, *p* = .609). We then conducted a between-subjects ANOVA to explore the effects of testimony type and gender on verdict confidence. There was no significant effect of gender or testimony-type on verdict confidence.

**Table 2:** ANOVA results for all main Dependent Variables in Study 1.

	Main Effect of Testimony-Type						Main Effect of Gender						Testimony-Type & Gender Interaction					
	*Study 1*			*Study 2*			*Study 1*			*Study 2*			*Study 1*			*Study 2*		
	*F*	*p*	ꞃ_p_^2^	*F*	*p*	ꞃ_p_^2^	*F*	*p*	ꞃ_p_^2^	*F*	*p*	ꞃ_p_^2^	*F*	*p*	ꞃ_p_^2^	*F*	*p*	ꞃ_p_^2^
Verdict Confidence	4.73	.009	.017	2.34	.097	.005	1.88	.172	.007	2.98	.085	.003	0.29	.75	.001	2.97	.052	.006
Defendant Blame	8.68	< .001	.031	0.63	.535	.001	13.69	< .001	.049	15.38	< .001	.016	0.80	.45	.003	0.23	.792	.000
Child Believable	7.67	.001	.028	1.84	.159	.004	14.57	< .001	.052	35.31	< .001	.036	4.59	.011	.017	1.18	.308	.002
Child Reliable	6.32	.002	.023	1.90	.150	.004	8.11	.005	.029	25.17	< .001	.026	0.46	.626	.002	0.41	.661	.001
Evidence Strength	125.90	< .001	.32	55.72	< .001	.105	0.32	.572	.001	17.54	< .001	.018	3.80	.024	.014	1.17	.310	.002
Evidence Credibility	137.35	< .001	.339	78.12	< .001	.141	0.21	.651	.001	5.90	.015	.006	4.79	.010	.018	1.18	.308	.002

#### Defendant blame

There was no significant effect of testimony type or gender on attributions of blame. There was a significant effect of gender. Females (*M* = 4.96, *SD* = 1.81) found the defendant to be more blameworthy than males (*M* = 4.51, *SD* = .1.73).

#### Child believability and reliability

There was no main effect of testimony-type on perceptions of child believability. A main effect of gender emerged, such that females (*M* = 5.66, *SD* = 1.23) found the child more believable than males (*M* = 5.14, *SD* = 1.36). Furthermore, there was no significant main effect of testimony type on perceptions of child reliability. There was a main effect of gender. Females (*M* = 5.08, *SD* = 1.47) found the child to be more believable than males (*M* = 4.59, *SD* = 1.58).

#### Evidence strength and credibility

We uncovered a significant main effect of testimony-type on perceptions of strength of the evidence. Evidence-based testimony was viewed as stronger than CSAAS, *p* < .001, *95% CI* (.54, 1.13), and the control expert testimony, *p* < .001, *95% CI* (1.10, 1.70). CSAAS expert testimony was seen as stronger than the control expert testimony, *p <* .001, *95% CI* (.29, .85). There was also a main effect of gender, such that females (*M* = 4.38, *SD* = 1.62) viewed the evidence to be stronger than males (*M* = 3.94, *SD* = 1.71).

Finally, we found a significant main effect of testimony-type on perceptions of credibility of the evidence. Evidence-based expert testimony was perceived as more credible than CSAAS, *p* < .001, *95% CI* (-1.43, -.85) and the control expert testimony, *p* < .001, *95% CI* (-1.86, -1.27). CSAAS expert testimony was seen as more credible than the control expert testimony, *p* = .01, *95% CI* (-.70, -.15). There was also a main effect of gender, such that females (*M* = 4.51, *SD* = 1.64) found the testimonies to be more credible than males (*M* = 4.25, *SD* = 1.69).

#### Exploratory mediation analysis

**Fig 3:**
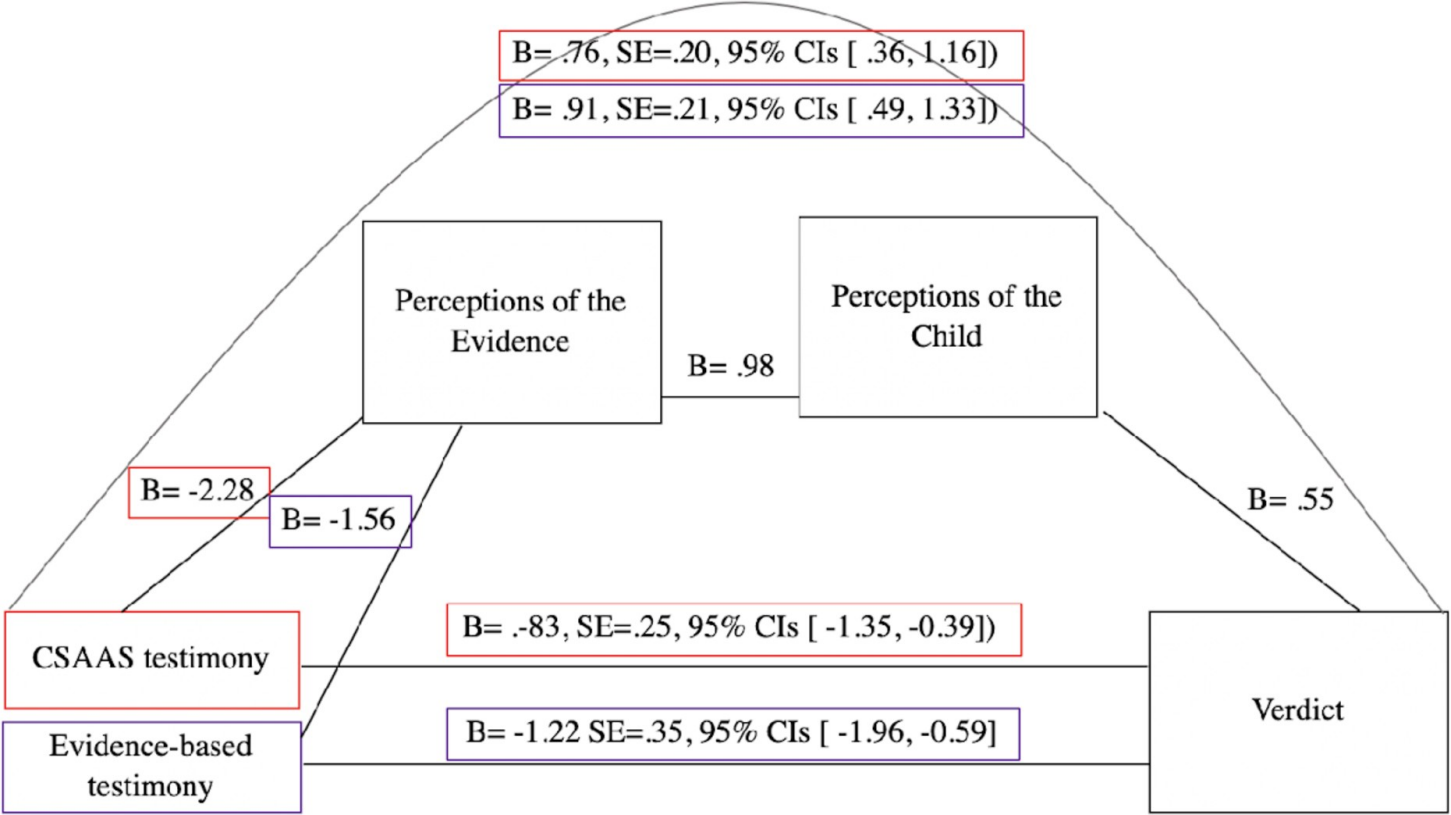
Exploratory mediation analysis for Study 2. Conceptual diagram of the exploratory serial mediation analyses in Study 2 depicting the relationship between Evidence-based Testimony and CSAAS testimony and verdicts when compared to Nonscientific experience-based testimony (the control).

Using Hayes PROCESS macro model 6, we explored whether a) strength of testimony predicted perceptions of the evidence, b) perceptions of the evidence predicted perceptions of the child, and c) perceptions of the child predicted verdicts. Model 6 tests this serial indirect effect of evidence strength on verdicts using 95% bias corrected bootstrap confidence intervals (10,000 samples). We combined child believability and child credibility to create the perceptions of child scale (Cronbach’s Alpha = .86). We further combined our credible and scientifically strong variables to create a perception of evidence scale (Cronbach’s Alpha = .88). We entered our perceptions of evidence scale followed by our perceptions of the child scale as serial mediators explaining the relationship between testimony condition and case verdicts. The indirect effect of testimony condition on verdicts through the two serial mediators was significant for both the Evidence-based testimony condition (IE = -1.22 SE = .35, 95% CIs [-1.96, -0.59]) and CSAAS testimony (IE =.-83, SE = .25, 95% CIs [-1.35, -0.39]) compared to the control (Nonscientific testimony). Specifically, testimony condition predicted perceptions of the strength of the evidence for both the Evidence-based testimony condition (B =.-2.28, SE = 0.18, 95% CIs [-2.63, -1.93) and CSAAS testimony (B =.-1.56, SE = 0.18, 95% CIs [-1.90, -1.21]) when compared to the control. In turn, perceptions of the strength of the evidence predicted perceptions of the child (B = .98, SE = .01 95% CIs [.96, 1.00). Finally, perceptions of the child (B = .55, SE = .14 95% CIs [.27, .82]) predicted case verdicts. See Fig 3 for visualization.

### Discussion

Consistent with Study 1, evidence-based testimony led to higher perceptions of credibility and scientific rigor of the evidence when compared to CSAAS testimony and the control testimony. This suggests that when presented alone, jurors’ ability to discern the strength of the evidence may be fairly robust. Evidence-based testimony also led to more guilty verdicts when compared to the control. Again, evidence-based testimony emerged as the only form of testimony consistently superior to the control in leading to guilty verdicts, yet it was not stronger than CSAAS testimony.

Exploratory mediation analyses provided an important insight into the paths by which jurors are applying testimony to their verdict preferences. Analyses revealed that testimony strength predicted a) perceptions of the evidence which in turn predicted b) perceptions of the child which then predicted c) verdicts. These findings provide some important preliminary insight into how jurors are applying the evidence given to them in testimony. The strength of the testimony is, logically, influencing jurors’ perceptions of the strength of the evidence. In turn, their perceptions of the strength of the case evidence influenced their perceptions of the child as believable and reliable. Their perceptions of the child as a believable and reliable witness then influenced their case verdicts. The serial process by which jurors are using the information given in testimony provides important insight into the decision-making process. Indeed, it seems that jurors’ perceptions of the child as a credible witness is an important factor influencing their case verdicts. The strength of testimony provided plays a role in shaping this perception. Yet this was only exploratory research–replication will be necessary to confirm the paths by which jurors are applying evidence to decision making.

Several of our findings were only present in Study 1. The direct effects of testimony on perceptions of the child, perceptions of the defendant, and subsequent verdict confidence were present only in Study 1. However, consistent across both studies, gender emerged as a predictor of perceptions of the child and defendant. There were large gender differences between Study 1 which was predominantly male, and Study 2, which was predominantly female. Both our own findings and those of previous studies suggest that gender is an important predictive factor in CSA cases [46]. It is possible then that these large gender differences may account for some of the variability in Study 1 and 2 results.

## General discussion

To date, there has been a lack of research systematically exploring the effects of expert testimony in child sexual abuse cases involving recantation. We were able to empirically test jury-eligible participants’ understanding of the quality of expert testimony presented to them and the subsequent effects on case verdicts. Importantly, we explored jurors’ perceptions of CSAAS and strong-evidence based testimony which differ meaningfully in scientific rigor. Based on a limited body of previous research, we expected jury-eligible participants to have some ability to discern the quality of evidence presented to them. We expected concrete implications for the perceptions of the victim, defendant, and resulting case judgments.

### Expert testimony strength and jurors’ assessments of recantation

Across Study 1 and Study 2 evidence-based expert testimony led to more guilty verdicts than our control testimony. In addition, participants were consistently able to distinguish strong expert testimony from both the control and CSAAS testimony, rating the evidence-based testimony as stronger and more credible. Yet, in Study 2, participants ratings of evidence strength and credibility for the control testimony and CSAAS testimony were indistinguishable. This suggests jurors have a robust ability to identify CSAAS as weak evidence–consistently weaker than evidence-based, and in some cases equally weak as intuition-based evidence.

Across both studies, jurors inconsistently differentiated between CSAAS and our control testimony when applying the evidence to perceptions of the child and defendant in the case. When participants did differentiate between evidence-based and CSAAS expert testimony, they often responded to CSAAS expert testimony similarly to how they responded to evidence-based testimony as evidenced by Study 1. Yet, Study 2 suggests this effect may not be consistent–a potential area for future research to establish the bounds of jurors’ application of evidence to case specific perceptions. Still, these findings suggest that while jury-eligible participants may understand evidence is weak, they are not systematically interpreting the evidence in a way that is reflected in case outcomes. While mock jurors have some ability to discern evidence strength and interpret the evidence appropriately, their ability to appropriately apply it is not robust or consistent [28]. Alternatively, it is possible that participants simply did not have enough information to make broader judgments related to the case.

While it seems evidence-based expert testimony can be effectively used to establish the child as a credible witness in CSA cases, recantation can be the result of either a false allegation or a true allegation. In some cases, children who recant are attempting to tell the truth and correct an untrue statement. Yet, this does not appear to be true of all recantations in cases as recantation is not a definitive indication that an allegation was false. Indeed, children who are young, have low maternal support, and stay in contact with their abuser are at a higher risk of recantation [7]. In these cases, where the prosecution believes recantation to be due to pressures on the child to take back a true allegation, expert testimony may be a useful avenue to rehabilitate a child’s credibility. A recantation should not immediately discredit a child, nor should it be ignored. Instead, as our research suggests, the quality of evidence presented in expert testimony may have some bearing on perceptions of the veracity of a child’s claims and the child’s credibility as a witness. Still these results need to be carefully interpreted in light of their limitations as this work lacks strong ecological validity. This is only the first step in exploring expert testimony in recantation cases–there is much work left to be done.

### General perceptions of CSA and recantation

This research provides some important insight to mock jurors general understanding of recantation. Mock jurors convicted the defendant an average of 62% of the time across both studies, most often rating the child as only “somewhat” believable and reliable. This is not surprising since cases where children provided inconsistent reports are significantly less likely to be viewed as having sufficient evidence to be brought to court [47, 48]. Despite mock jurors indicating that they believe recantation happens in about 30% of cases, they did not view children who recanted as strongly reliable and believable. We randomized the presentation of these estimates to occur either before or after case descriptions, suggesting these are an accurate reflection of jury-eligible adult’s estimates of recantations rates. Interestingly, these estimates are very close to Malloy and collegues (2007) estimates of recantation rates suggesting participants make have a gist level understanding of recantation rates.

### Limitations and future directions

This study has limitations. Primarily, this is an online examination of jury-eligible adults, not jurors in a real case. While neither study involved jury deliberation, research involves methodological choices constrained by resources and validity tradeoffs. Reviews have found that jury researchers value jury instructions more than deliberation [49]. Though not ideal, we can make valuable advances in our understanding of juror behavior and decisions without jury deliberation. We can understand what jurors are bringing to deliberation and their perceptions of the evidence presented to them. In addition, we used a simplified and summarized case structure as opposed to a full case. In a true case, expert testimony is much longer, and cross-examination is more thorough. Furthermore, jurors hear other evidence in the case which may exert an influence on their final case judgments. This may also give jurors a better opportunity to critically analyze the quality of the evidence presented to them. However, this is a first attempt to get a sense of how jurors might respond, and the first study to provide empirical data on the effectiveness of expert testimony in recantation cases. Furthermore, our separation of the conditions is an idealized difference that doesn’t completely reflect the real world. We have conducted this research in a non-ecological setting, suggesting the generalizability may be limited. However, this is an issue with experimental research generally—the tension between laboratory research and external validity. This research is the first attempt to explore expert testimony in recantation cases, where more ecologically valid work will need to be done to truly generalize to real world cases. These findings still provide valuable insight into the role of quality of expert testimony in mock-juror decision making.

In addition, it is possible that the gender effects we uncovered are a result of the composition of the vignettes. That is, a male perpetrator and a female victim may evoke more sympathy from women due to the gender dynamic represented. Finally, our findings in Study 1 did not perfectly replicate in Study 2. There are several explanations for this. As evidenced by the predictive role of gender in all of our results, individual differences play a large role in predicting CSA case outcomes. The repeated-measure design of Study 1 allowed us to account for these individual differences–something we could not do in Study 2. As we cannot parse apart the true effect and the noise of individual response patterns in Study 2, it is possible this is accounting for some of our null findings. Still, it is possible that the results of Study 1 were a product, in part, of demand characteristics due to the repeated-measures nature of our design. Yet, our core findings on the effect of testimony strength on verdict, scientific credibility, and evidence strength replicated in Study 2 with a much larger sample, suggesting these findings are robust and useful for out of sample predictions.

Despite these stated limitations, our study design has numerous advantages over a true case analysis. We used a sample of jury eligible adults and used manipulation checks to help ensure thoughtful participation. We were able to randomize ages, names, expert qualifications, and race in ways that would not systematically affect our results–factors which are likely to impact the case outcome in important ways but cannot be controlled for in a real case. The abridged versions we used also allowed us to narrow jurors’ attention on just our factors of interest, which may allow for an easier detection of the effect of testimony-type. Future research could be used to apply these experimental findings to a real case context with real expert testimony.

### Practice implications, public policy implications, and conclusions

This line of research helps to advance knowledge on how jury-eligible participants understand the scientific credibility of research in a context where expert testimony is particularly influential–CSA cases. This research supports modern scientific evidence as a potentially useful tool for assisting jurors in understanding the evidence in CSA cases; researchers will be need to continue to confirm the role of quality of evidence in CSA cases. It seems jurors’ misconceptions about CSA may be difficult to overcome and expert testimony only consistently affected verdicts, and inconsistently affected perceptions of the defendant and alleged victim. This line of research suggests a need to explore the role of testimony in these cases and how to better correct jurors’ misconceptions which seem to be strongly influenced by individual differences. Historically, experts have relied on CSAAS as an explanation for children’s disclosure behaviors, despite strong evidence that CSAAS was not based on sound methodology or empirical evidence. Still, modern empirical research has supported many of Summitt’s original claims [10]. What do these findings mean for courts grappling with recantation cases and expert testimony? We suggest that experts testify in CSA cases involving recantation with strong evidence-based testimony to help jurors understand recantation, allowing jurors to carefully evaluate a child’s allegation in these types of cases.
